# Prion protein lacks robust cytoprotective activity in cultured cells

**DOI:** 10.1186/1750-1326-3-11

**Published:** 2008-08-21

**Authors:** Heather M Christensen, David A Harris

**Affiliations:** 1 Department of Cell Biology and Physiology, Washington University School of Medicine, 660 South Euclid Avenue, St. Louis, MO 63110, USA

## Abstract

**Background:**

The physiological function of the cellular prion protein (PrP^C^) remains unknown. However, PrP^C ^has been reported to possess a cytoprotective activity that prevents death of neurons and other cells after a toxic stimulus. To explore this effect further, we attempted to reproduce several of the assays in which a protective activity of PrP had been previously demonstrated in mammalian cells.

**Results:**

In the first set of experiments, we found that PrP over-expression had a minimal effect on the death of MCF-7 breast carcinoma cells treated with TNF-α and *Prn-p*^*0/0 *^immortalized hippocampal neurons (HpL3-4 cells) subjected to serum deprivation. In the second set of assays, we observed only a small difference in viability between cerebellar granule neurons cultured from PrP-null and control mice in response to activation of endogenous or exogenous Bax.

**Conclusion:**

Taken together, our results suggest either that cytoprotection is not a physiologically relevant activity of PrP^C^, or that PrP^C^-dependent protective pathways operative *in vivo *are not adequately modeled by these cell culture systems. We suggest that cell systems capable of mimicking the neurotoxic effects produced in transgenic mice by N-terminally deleted forms of PrP or Doppel may represent more useful tools for analyzing the cytoprotective function of PrP^C^.

## Background

Prion diseases, also known as transmissible spongiform encephalopathies, are fatal neurodegenerative disorders that occur when the normal, cellular prion protein (PrP^C^) is converted into a conformationally altered isoform (PrP^Sc^) that is self-propagating and infectious [1,2]. While the properties of PrP^Sc ^and its role in the disease process have been extensively characterized, the normal physiological function of PrP^C ^has yet to be resolved. Mice genetically lacking PrP^C ^exhibit no gross anatomical or developmental abnormalities, and have been largely uninformative for deducing a physiological function of PrP^C ^[3-5]. Several potential functions have been proposed for PrP^C^, including protection from apoptosis and oxidative stress, maintenance of synaptic integrity, regulation of copper metabolism, cell signaling, and cell adhesion (reviewed in [6]). Whatever the physiological function of PrP^C^, it has become increasingly clear that expression of PrP^C ^is necessary to mediate the toxicity induced by PrP^Sc ^[7-9]. Therefore, determining the normal function of PrP^C ^will likely provide important insight into the neurotoxic mechanisms underlying prion diseases.

The most compelling evidence for a functional activity of PrP^C ^comes from studies in mice expressing either certain N-terminally deleted forms of PrP or the PrP paralog, Doppel (Dpl). Several different deletions that encompass a highly conserved sequence of 21 amino acids in the central region of PrP all cause a spontaneous neurodegenerative illness when expressed in the brains of transgenic mice [10-12]. A neurodegenerative phenotype is also observed in mice in which Dpl, a PrP paralog that is normally expressed in testes and structurally resembles the C-terminal globular domain of PrP, is ectopically expressed in the brain [13-16]. Strikingly, neurodegeneration induced by deleted PrP molecules and Dpl is reversed by co-expression of wild-type PrP [10-12,17]. These results have been interpreted to mean that deleted PrP and Dpl activate a common neurotoxic pathway, and that this pathway is suppressed by the presence of wild-type PrP.

The dramatic rescuing effect by wild-type PrP in these transgenic mice raises the possibility that PrP^C ^possesses a generalized neuroprotective activity that could counteract the effects of other toxic stimuli [6,18]. One kind of evidence consistent with this idea derives from studies of cultured neurons or mice that lack PrP expression. For example, it has been reported that neurons cultured from *Prn-p*^*0/0 *^mice are more susceptible than wild-type neurons to several kinds of oxidative stress including exposure to xanthine oxidase, hydrogen peroxide, and copper ions [19]. There is also evidence that PrP^C ^may play a protective role *in vivo *during focal cerebral ischemia or traumatic brain injury. PrP^C ^expression levels increase after these kinds of injury and lesion size is larger in *Prn-p*^*0/0 *^compared to wild-type mice [20,21]. Retinal photoreceptors from *Prn-p*^*0/0 *^mice have also been reported to be more susceptible to light-induced apoptosis [22].

Several *in vitro *systems have also been described in which increased PrP expression has been found to exert a protective effect against apoptotic insults [23]. In one such system, human fetal neurons in culture were induced to undergo apoptosis by microinjection of a plasmid encoding the pro-apoptotic protein, Bax. Co-injection of a PrP-encoding cDNA efficiently prevented Bax-induced neuronal death [24]. This protective effect has been attributed to the presence of a cytosolic form of PrP, which is thought to inhibit conformational activation of Bax [25-27]. In a second example, serum deprivation of two kinds of immortalized hippocampal cell lines (HpL and NpL) derived from *Prn-p*^*0/0 *^mice triggered an apoptotic response that was rescued by transfection with a PrP-encoding plasmid [28-30]. Over-expression of PrP^C ^has also been reported to render MCF-7 breast carcinoma cells resistant to apoptosis induced by TNF-α, TRAIL, and Bax [25,27,31,32]. PrP expression also correlated with increased resistance of SGC7901 gastric carcinoma cell lines to several chemotherapeutic agents [33]. Finally, work in our own laboratory has shown that expression of mammalian PrP markedly protects *S. cerevisiae *from Bax-induced cell death [34]. Although these studies utilize a variety of cell types and a range of different stimuli to induce cell death, it is possible that the protective activity of PrP observed in each case reflects a common, underlying molecular mechanism. However, the nature of this mechanism remains unknown.

In this study, we have re-examined the cytoprotective activity of PrP in three different mammalian cell systems similar to those that have been previously published. We first attempted to reproduce the rescue effect of PrP in TNF-α-treated MCF-7 cells and serum-deprived HpL cells. To examine the protective activity of PrP in primary neurons, we assessed the ability of PrP to inhibit apoptosis of cerebellar granule neurons induced by expression of exogenous Bax and by activation of endogenous Bax following potassium and serum withdrawal [35]. While our results demonstrate a weak cytoprotective effect by PrP in each of these systems, the degree of protection was considerably less than in previously published studies. The modest cytoprotective effects observed here lead us to consider whether PrP possesses any physiologically relevant neuroprotective activity, and if so, whether the toxic stimuli used did not effectively activate the relevant PrP-dependent pathways. This work has important implications for the design of experimental strategies aimed at uncovering the mechanisms of PrP^C ^cytoprotection.

## Results

### Expression of PrP in MCF-7 cells weakly suppresses death induced by TNF-α

**Figure 1:**
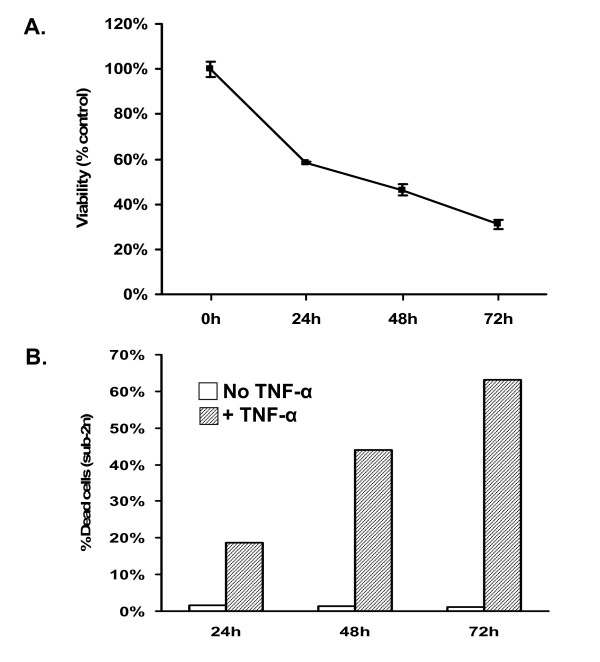
**Untransfected MCF-7 cells undergo cell death following treatment with recombinant TNF-α**. (A) MCF-7 cells were treated with 100 ng/ml TNF-α, and cell viability was assessed using the MTT assay. Absorbance values are expressed as a percentage of those in non-treated control samples at each timepoint. Error bars are derived from triplicate wells of one representative experiment. (B) Flow cytometry analysis of untreated or TNF-α-treated MCF-7 cells. Cells were trypsinized, fixed in ethanol, and incubated with propidium iodide prior to analysis by flow cytometry. 10,000 cells were counted for each condition. Data are expressed as the percentage of cells containing sub-2n DNA content in one representative experiment.

To test the observation that PrP^C ^rescues MCF-7 human breast carcinoma cells from TNF-α-mediated cell death [31], we first determined the sensitivity of MCF-7 cells to TNF-α treatment using two different assays: MTT dye reduction, and flow cytometry after propidium iodide staining to measure the proportion of cells with sub-2n DNA content. Treatment of untransfected MCF-7 cells with 100 ng/ml TNF-α diminished cell viability over time, with the MTT signal reduced to ~30% after 72 hrs (Figure 1A). A similar time course of cell death was observed by flow cytometry, with the proportion of cells containing sub-2n DNA reaching ~65% by 72 hrs (Figure 1B). These data confirm the susceptibility of MCF-7 cells to cell death induced by treatment with TNF-α in the absence of exogenous PrP expression.

**Figure 2:**
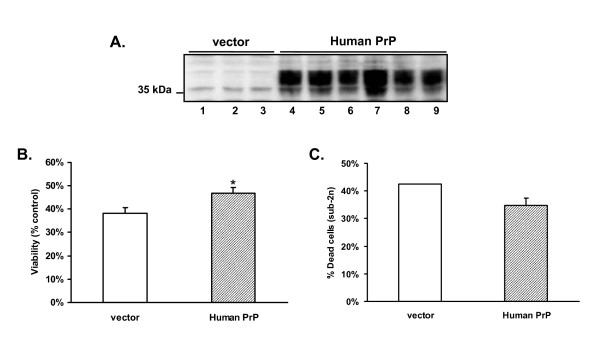
**Expression of human PrP mildly suppresses TNF-α-mediated death of MCF-7 breast carcinoma cells**. (A) PrP expression was assessed by western blotting of three pools of vector-transfected MCF-7 cells (lanes 1–3) and six pools of MCF-7 cells transfected with a human PrP plasmid (lanes 4–9). (B) Pools of MCF-7 cells were treated with 100 ng/ml of recombinant TNF-α for 43 h. Cell viability was determined by the MTT assay. Absorbance values are expressed as a percentage of those in non-treated control samples. Data represent the mean ± SEM from 3 vector-transfected pools and 6 PrP-transfected pools. *p = 0.0042, human PrP vs. vector. (C) Pools of MCF-7 cells were treated with 100 ng/ml of recombinant TNF-α for 43 h. Cell viability was determined by flow cytometry after staining with propidium iodide. 10,000 cells were counted for each condition, and data are expressed as the percentage of cells containing sub-2n DNA. Data are derived from 4 MCF-7(hPrP) pools (mean ± SEM) and one vector control pool.

To test the rescuing effect of PrP, we generated pools of MCF-7 cells that were transfected either with an empty vector, or with vector encoding human PrP. We analyzed three independent pools of vector-transfected cells which express low levels of endogenous PrP, and six pools of cells expressing high levels of transfected PrP (~20–25-fold over endogenous) (Figure 2A). Each pool was treated with 100 ng/ml TNF-α for 43 hrs, and then assayed by MTT and flow cytometry. We observed that the PrP-expressing pools displayed a small, but statistically greater viability compared to vector-transfected pools based on the MTT assay (Figure 2B): 47% (PrP) vs. 38% (vector) (p = 0.0042). Less cell death was also observed in PrP-expressing cells than in vector controls, as measured by the proportion of cells with sub-2n DNA content (Figure 2C): 35% (PrP) vs. 42% (vector).

### PrP weakly suppresses death of immortalized hippocampal neurons following serum deprivation

**Figure 3:**
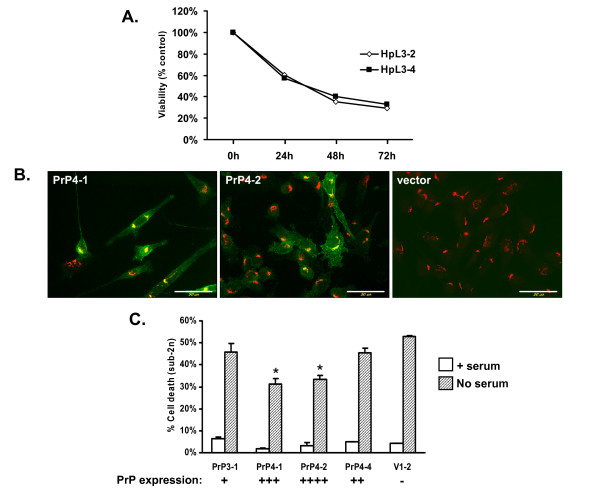
**Stable expression of PrP weakly suppresses death of immortalized *Prn-p*^*0/0 *^hippocampal neurons after serum deprivation**. (A) Two untransfected lines of *Prn-p*^*0/0 *^HpL cells (3–2 and 3–4) were subjected to 72 hours of serum deprivation. Cell viability was determined by MTT assay. Absorbance values are expressed as a percentage of those in non-treated control samples at each timepoint. Data are from one representative experiment. (B) Immunofluorescence analysis of mouse PrP expression in two PrP-expressing lines of HpL3-4 cells (4-1 and 4-2), and one vector-transfected line. PrP was detected by staining with the PrP antibody (green). Cells were counterstained with giantin, a marker for the Golgi apparatus (red). PrP expression was detectable on the cell surface and in the Golgi apparatus. Bar = 50 μm. (C) HpL3-4 cell lines were subjected to 96 hours of serum deprivation, after which cell viability was determined by flow cytometry after propidium iodide staining. 10,000 cells were counted for each condition. Data are expressed as the percentage of cells containing sub-2n DNA content, and represent the mean ± SEM from three independent experiments. *p < 0.01, PrP vs. vector. Relative PrP expression for each cell line is indicated below each set of bars by the number of "+" symbols.

PrP has also been reported to exert a protective activity in immortalized hippocampal cell lines derived from *Prn-p*^*0/0 *^mice (HpL cells) subjected to serum deprivation [29]. We obtained two independently generated HpL cell lines (HpL3-2 and HpL3-4) and tested their susceptibility to serum withdrawal. Both lines underwent cell death over a comparable time course, with ~30% of the cells remaining viable after 72 hrs (Figure 3A).

We then generated stably transfected lines of HpL3-4 cells expressing wild-type mouse PrP, and tested their susceptibility to cell death following serum deprivation. We analyzed four independent lines that expressed differing levels of PrP, based on immunofluorescence staining (Figure 3B). PrP-expressing and vector-transfected lines were deprived of serum for 96 hrs, and cell death was measured by flow cytometry (Figure 3C). Data was normalized relative to untreated controls. We observed a modest rescuing effect that was correlated with the level of PrP expression level. Two lines expressing the highest levels of PrP displayed a reduced level of cell death that was statistically significant relative to a vector control (PrP4-1, p = 0.0068; PrP4-2, p = 0.0022). All PrP-expressing clones showed less cell death than a vector control clone (V1-2), in which > 50% of the cells were dead by 96 hrs.

### HpL cells do not express neuronal markers or Doppel

Since PrP did not dramatically rescue HpL cells from serum deprivation as previously reported [29], we sought to confirm the identity of these cells by analyzing their expression of various neuronal and astrocytic markers using immunofluorescence and quantitative RT-PCR (Table 1). As previously reported [28,29], neither the HpL3-2 nor HpL3-4 lines expressed appreciable levels of GFAP. Surprisingly, however, we did not detect expression of the neuronal markers NeuN, MAP2, or the 68 K neurofilament light-chain subunit (NF-L). These cells were previously reported to express NF-L [29]. Using the same antibodies, we previously observed high levels of the neuronal and astrocytic markers in brain (Table 1) [36].

**Table 1:** Analysis of marker proteins in HpL3-2 and HpL3-4 cells by immunofluorescence and quantitative RT-PCR.

**SAMPLE**	**IMMUNOFLUORESCENCE**			**qRT-PCR**		
		
	**NeuN**	**MAP2**	**GFAP**	**NF-L**	**GFAP**	**Doppel**
HpL3-2	-	-	-	-	-	-
HpL3-4	-	-	-	-	-	-
Brain	+ *	+ *	+ *	+	+	N/A
Testes	N/A	N/A	N/A	N/A	N/A	+

* Immunofluorescence detection of NeuN, MAP2, and GFAP in brain sections was performed previously [36].

HpL cells are derived from hippocampal neurons cultured from Rikn *Prn-p*^*0/0 *^mice, in which an intergenic splicing event caused Doppel to be ectopically expressed in brain under control of the PrP promoter [37,38]. Unexpectedly, quantitative RT-PCR and immunofluorescence analysis failed to detect expression of Doppel in the HpL3-2 or HpL3-4 cells (Table 1).

### PrP does not significantly reduce death of cerebellar granule neurons (CGNs) induced by exogenous Bax

**Figure 4:**
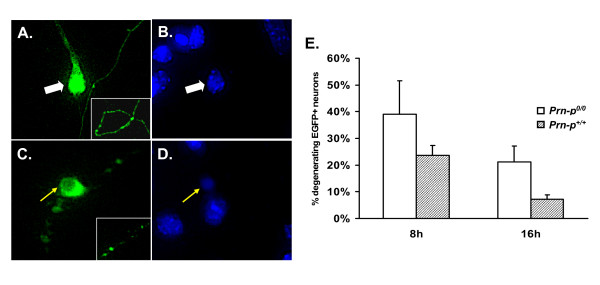
**PrP does not significantly reduce death of cerebellar granule neurons induced by exogenous Bax**. (A-D) Representative fluorescence images of cerebellar granule neurons from *Prn-p*^*0/0 *^mice transfected with plasmids encoding EGFP and mouse Bax. Four days after transfection, cultures were stained with DAPI, and EGFP-positive neurons were scored as healthy (A, B) or apoptotic (C, D) based on morphological criteria. These criteria included the distribution of EGFP in the soma and neurites, and the appearance of DAPI staining in the nucleus (see text). (E) CGNs cultured from *Prn-p*^+/+ ^or *Prn-p*^*0/0 *^pups were transfected with plasmids encoding Bax and EGFP. Cultures were fixed and stained with DAPI either 8 or 16 hours after transfection, and scored for apoptotic morphology. Data represent the mean ± SEM from at least three independent experiments. The difference between *Prn-p*^*0/0 *^and *Prn-p*^+/+ ^neurons did not reach statistical significance at either time point (p > 0.05).

To test the hypothesis that PrP can inhibit Bax-induced cell death, we transiently transfected a cDNA encoding mouse Bax into CGNs cultured from either *Prn-p*^+/+ ^or *Prn-p*^*0/0 *^mice. A separate plasmid encoding EGFP was co-transfected into the neurons as a marker of transfected cells. In healthy neurons, EGFP fluorescence was distributed evenly throughout the soma (Figure 4A, white arrow) and continuously along the neurites (Figure 4A, inset). By DAPI staining, the nucleus of healthy cells was relatively large, and filled with multiple nucleoli (Figure 4B, white arrow). Cell death induced by Bax resulted in characteristic morphological changes that were easily visualized by EGFP and DAPI fluorescence. Dying granule cells displayed a soma that was shrunken (Figure 4C, yellow arrow), as well as coalescence of EGFP into large, unconnected aggregates or beads along the neuritic process, indicative of neuritic degeneration (Figure 4C, inset). Co-staining with DAPI also revealed pyknotic nuclei (Figure 4D, yellow arrow).

Eight hours after transfection of *Prn-p*^*0/0 *^cultures with the Bax plasmid, 39% of EGFP-positive neurons were degenerating based on morphological criteria (Figure 4E). Cultures from *Prn-p*^+/+ ^mice displayed a reduced percentage of apoptotic neurons (23%), although this difference was not statistically significant (p = 0.3038). By 16 hrs after transfection, *Prn-p*^*0/0 *^cultures contained more degenerating CGNs than *Prn-p*^+/+ ^cultures, a difference that approached but did not reach statistical significance (21% vs. 7%, respectively; p = 0.0665). Cultures derived from either *Prn-p*^*0/0 *^or *Prn-p*^+/+ ^mice transfected with empty vector (without Bax) contained < 2% apoptotic CGNs.

### CGNs expressing PrP are slightly more resistant to cell death induced by potassium/serum deprivation

**Figure 5:**
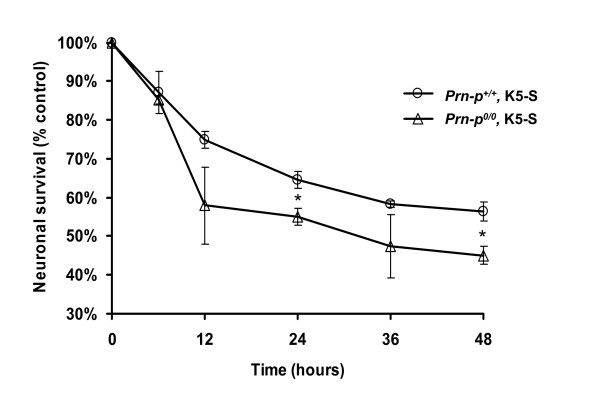
**CGNs expressing PrP are slightly more resistant to cell death induced by potassium/serum deprivation**. CGNs cultured from *Prn-p*^*0/0 *^and *Prn-p*^+/+ ^mice were cultured in K25+S medium for 7 days, after which they were transferred to K5-S medium for the designated times. Cell viability was assessed by measurement of calcein fluorescence on a microplate fluorimeter. Fluorescence values in K5-S medium are expressed as a percentage of those in K25+5 medium at each time point. Data represent the mean ± SEM for at least two independent experiments. * p < 0.05, *Prn-p*^+/+ ^vs. *Prn-p*^*0/0*^.

To determine whether PrP protects neurons from death due to activation of endogenous Bax, CGNs from wild-type and *Prn-p*^*0/0 *^mice were transferred to medium containing reduced potassium and no serum. To improve viability, CGNs are routinely cultured in the presence of serum under depolarizing conditions with elevated potassium (25 mM). Reducing potassium to 5 mM and removing serum causes the cells to undergo Bax-dependent apoptosis [39,40]. Using calcein AM fluorescence to detect viable neurons, we observed that incubation in low-potassium medium without serum (K5-S) gradually reduced the viability of *Prn-p*^+/+ ^CGNs to 56% of the value for cells maintained in control medium (K25+S) after 48 hrs (Figure 5, open circles). CGNs cultured from *Prn-p*^*0/0 *^mice consistently followed a slightly faster reduction in viability, with 45% of the neurons surviving after 48 hrs (Figure 5, open triangles). The difference in viability between the two types of neurons was statistically significant at 24 and 48 hrs (p = 0.0358 and 0.0302, respectively). These data demonstrate that endogenous PrP expression slightly reduces, but does not prevent, Bax-dependent cell death of CGNs induced by lowering extracellular potassium and removing serum.

## Discussion

In this study, we tested the ability of PrP to protect cultured cells from several kinds of cytotoxic insults using assays similar to those that have previously been published by other investigators. We examined the effect of PrP expression on the viability of MCF-7 breast carcinoma cells treated with TNF-α, HpL3-4 immortalized hippocampal neurons deprived of serum, and cultured cerebellar granule neurons induced to undergo two kinds of Bax-dependent apoptosis. In contrast to previously published reports, we failed to detect a robust protective effect of PrP in any of these assays. These results have led us to critically reevaluate the function of PrP as a cytoprotective molecule.

### The protective activity of PrP in MCF-7 and HpL3-4 cells is more modest than previously described

In a DNA microarray analysis, Diarra-Mehrpour et al. [31] found that PrP expression was up-regulated 17-fold in a sub-line of MCF-7 cells that was resistant to TNF-α-induced apoptosis. These investigators then showed that adenovirally-mediated over-expression of PrP in the parental TNF-α-sensitive MCF-7 cell line conferred resistance to TNF-α, an effect that correlated with reduced cytochrome c release from mitochondria. In their experiment, ~80% of PrP-expressing cells remained viable after TNF-α treatment compared with ~30% for untransduced cells. In our hands, in contrast, over-expression of PrP in MCF-7 cells by a similar amount had a much more modest effect, increasing viability after TNF-α treatment from 38% to 47% (Figure 2B).

Although a number of published studies have utilized HpL3-4 cells to demonstrate a neuroprotective activity of PrP [28-30,37,41-46], only two report quantitative data concerning the ability of PrP to rescue these cells from serum deprivation [41,42]. In one of these studies [42], stable expression of PrP in HpL3-4 cells was reported to reduce cell death by 80% relative to vector controls based on LDH assays of cell death. However, the LDH data were normalized to the values for vector-transfected cells after 24 hours of serum deprivation, so it is not possible to gauge of the absolute amount of cell death occurring in these experiments. In the second study [41], serum deprivation reduced the viability of vector-transfected cells by only a modest amount (to 75% of the value for untreated cells) and PrP expression restored viability to nearly 100% after 24 hours. In our experiments, stable expression of PrP in several independent HpL3-4 cell lines produced only a small reduction in cell death, amounting to < 20%, even in lines expressing the highest levels of PrP (Figure 3C).

It is unclear what factors account for the discrepancies between our results and those reported in previous studies. In the case of MCF-7 cells, one possible reason may be a difference in the methods used to introduce exogenous PrP: stable transfection in our experiments vs. adenoviral transduction in the study of Diarra-Mehrpour et al. [31]. However, both methods achieved similar levels of over-expression (~25-fold). Alternatively, genetic variation between different lines of MCF-7 cells may play a role. Susceptibility of MCF-7 cells to TNF-α treatment is greatly influenced by genetic factors, leading to cell line variants with differing sensitivities to TNF-α-induced apoptosis [47,48]. Signaling molecules influencing the response to TNF-α also differ between variants of MCF-7 cells, including PKCε, JNK, p53, and NF-κB [49-53]. The diversity of factors that affect TNF-α-mediated cell death in MCF-7 cells suggest that the previously described protective effect by PrP may be specific for a particular strain variant and may not be generally reproducible in other cell types.

Similar genetic changes may have occurred in HpL3-4 cells, since we did not detect expression of NF-L or Doppel by quantitative RT-PCR (Table 1). The absence of NF-L expression in the HpL3-4 and HpL3-2 cells suggests that they may have lost their neuronally differentiated characteristics, which may be required for the rescue activity.

### PrP does not strongly rescue cultured neurons from Bax-dependent apoptosis

We have proposed several possible mechanisms to explain how PrP could exert a protective effect against Bax-mediated cytotoxic insults [6]. PrP may act on death receptors at the cell surface, it may influence the activity of pro- and anti-apoptotic molecules by physical interaction or via signal transduction pathways, or it could work within intracellular organelles to influence cell death pathways. To test whether PrP could modulate Bax-dependent pathways, we utilized cultured CGNs in an attempt to recapitulate the dramatic rescue activity that was described in analogous experiments on human primary neurons [24]. We chose to utilize CGNs for two reasons. First, CGNs undergo Bax-dependent apoptosis in response to reduced extracellular potassium and serum deprivation, and this process has been analyzed extensively to gain insight into the mechanisms of programmed cell death [40,54]. Second, CGNs have been used by others to demonstrate a neuroprotective activity of PrP against Dpl [55,56].

In contrast to the previous study [24], we failed to observe a robust protective effect of PrP against apoptosis induced either by ectopic expression of Bax or by activation of endogenous Bax via potassium depletion and serum deprivation. In these experiments, the presence of PrP enhanced cell viability by at most 15%. The reasons for this discrepancy are unclear, but differences in neuronal populations (cortical versus cerebellar), species (human versus mouse), or PrP expression level may play a role. With regard to the last point, Bounhar et al. utilized microinjection of a cDNA-encoding plasmid in order to boost PrP expression in neurons that presumably already contained endogenous PrP, although no data were presented on the degree of over-expression achieved [24]. In contrast, we compared neurons completely lacking PrP to those containing normal endogenous levels. It is possible that Bax rescue activity is only observed with supraphysiological expression levels of PrP.

The role of Bax in the physiological activity of PrP^C ^remains uncertain, with some studies suggesting a functional connection between the two proteins and others arguing against it. Our own studies [34] as well as those of others [57] demonstrate that *S. cerevisiae *yeast cells expressing mammalian PrP are protected from Bax-induced cell death. In addition, PrP expression was shown to reduce cell death in MCF-7 cells by inhibiting a pro-apoptotic conformational change in Bax [25]. Deletion of the Bax gene rescues neuronal loss in Tg(PG14) mice expressing a disease-associated mutant PrP with impaired neuroprotective activity [58,59] but does not prevent neuronal death in transgenic mice expressing PrPΔ105-125 or PrPΔ32-134 [60]. Bax deletion has no effect on the disease course in scrapie-infected mice [61,62]. Since PrP is largely present on the cell surface and Bax is localized to the cytosol, interaction between the two proteins, if it occurs, must either be indirect or else involve rare cytosolic forms of PrP [26,27]. Relevant to this issue, there is evidence that cytosolic PrP physically associates with the Bax antagonist, Bcl-2 [63], but not with Bax itself [25].

### Cell culture models to investigate the cytoprotective activity of PrP

In a number of cell types, expression of PrP seems to exert a protective effect against several different toxic insults [6,23]. Although we failed to observe a robust protective activity when we re-examined three of the published systems, we did note that the presence of PrP was associated with a small improvement (< 20%) in cell viability in some of our experiments. Whether these effects observed in cell culture experiments reflect a physiologically relevant activity of PrP^C ^remains to be determined. It is possible that they represent *in vitro *artifacts due to PrP over-expression or other factors. Alternatively, PrP^C ^may possess a cytoprotective activity *in vivo *that is not easily reproduced in cell culture models. This might be the case if the cellular stresses applied experimentally do not adequately mimic those operative in brain tissue, or because cultured cells lack some of the relevant PrP-dependent death pathways.

At present, the most compelling evidence for a role of PrP in cytoprotective and cytotoxic phenomena comes from mice expressing Dpl or PrP forms harboring deletions that span residues 105–125 [10-12,15]. These molecules produce a dramatic neurodegenerative phenotype that is dose-dependently suppressed by co-expression of wild-type PrP. Therefore, it would seem that cell culture systems capable of reproducing this phenomenon would be the most useful tools for investigating the toxic and protective activities of PrP^C^. Several reports have appeared in which expression of Dpl or deleted PrP forms in cultured cells impairs viability, with co-expression of wild-type PrP suppressing this effect [37,55,56,64]. Further development of these and other cell models will greatly aid in deciphering the physiological function of PrP^C^, and how it might be subverted during the disease process.

## Conclusion

In this study, we investigated the cytoprotective activity of PrP^C ^in several mammalian cell culture systems. In each system tested, expression of wild-type PrP elicited a modest protective effect, although less than in previously published studies. We conclude that the cytoprotective activity of PrP^C ^observed *in vivo *is not easily recapitulated *in vitro*. Either the cellular stresses employed do not engage a PrP^C^-dependent pathway, or cytoprotection is not a physiologically relevant activity of PrP^C^.

## Methods

### Cell lines

MCF-7 and HpL cells were cultured in DMEM supplemented with 10% FCS, 2 mM glutamine, and penicillin/streptomycin, and were maintained in a humidified incubator at 37°C in 5% CO_2_.

MCF-7 cells were transfected with a pcDNA3 plasmid encoding wild-type human PrP using Lipofectamine 2000 (Invitrogen, Carlsbad, CA) according to the manufacturer's protocol. Forty-eight hours later, cells were split into 700 μg/ml G418 (Tissue Culture Support Center, Washington University in St. Louis) for two weeks. Cells were harvested as pools and maintained in 300 μg/ml G418. PrP expression was detected by Western blotting of cell lysates using the monoclonal anti-PrP antibody, 3F4 (1:1000) [65].

HpL3-4 cells were transfected with wild-type mouse PrP cDNA contained in a pcDNA3.1(+)/hygro vector (Invitrogen). After transfection with Lipofectamine 2000, cells were selected in media containing 200 μg/ml hygromycin B (Invitrogen). Upon selection of stable lines, cells were maintained in 100 μg/ml hygromycin B.

PrP expression was assessed by Western blotting using the monoclonal 3F4 antibody (MCF-7 cells), or by immunofluorescence staining (HpL cells, see below).

### Immunofluorescence staining of HpL cells

HpL cells were fixed with 4% paraformaldehyde, permeabilized with 0.5% Triton-X100 in PBS, and blocked in 5% goat serum in PBS. Cells were incubated with the monoclonal anti-PrP antibody 8H4 [66], and co-stained with antibodies to the Golgi marker, giantin (1:1000; Covance, Berkley, CA). In some experiments, cells were stained with antibodies to GFAP (1:1000; Dako, Carpinteria, CA), MAP-2 (1:1000; Sigma, St. Louis, MO) and NeuN (1:1000; Chemicon, Temecula, CA). After incubation with primary antibodies, cells were treated with Alexa Fluor secondary antibodies (Alexa-488 goat anti-mouse IgG and Alexa-594 goat anti-rabbit IgG) prior to imaging using a Nikon OptiPhot-2 microscope and MetaMorph imaging software.

### Measurement of death and viability of MCF-7 and HpL cells

Stably transfected pools of MCF-7 cells expressing human PrP were treated with 100 ng/ml recombinant TNF-α (PeproTech, Rocky Hill, NJ) in growth medium for 43 hours. Stimulation of cell death by serum deprivation in HpL3-2 and HpL3-4 cells was performed as previously described [29].

For MTT viability assays, medium was replaced with 0.32 mg/ml MTT (3-(4,5-dimethyl-2-thiazolyl)-2,5-diphenyl-2H-tetrazolium bromide; Sigma, St. Louis, MO) in Locke's buffer for 30–60 minutes. Cells were solubilized with DMSO and the absorbance was read at 575 nm in a microplate reader (Bio-Tek, Winooski, VT). Experimental values were expressed as a percentage of the absorbance values in non-treated control samples.

For flow cytometry analysis, cells were washed once with PBS prior to trypsinization and fixation in 70% cold ethanol. After fixation, cells were washed in PBS/1% BSA, pelleted, and resuspended in propidium iodide (PI) working solution containing 30 μg/ml PI (Sigma) and 0.25 mg/ml RNAse A (Qiagen, Valencia, CA) in PBS/1% BSA. The volume of PI working solution was normalized for cell number. Flow cytometry was performed on a FACSCalibur flow cytometer (BD Biosciences, San Jose, CA), and the data was analyzed using CELLQUEST analysis software (BD Biosciences).

### Quantitative RT-PCR analysis

mRNA was purified from HpL3-4 and HpL3-2 cells and wild-type mouse brain and testes using the RNAwiz reagent (Ambion, Austin, TX). cDNA was generated, and expression of GFAP, NF-L, and Doppel was analyzed by quantitative RT-PCR using an ABI-PRISM 7000 Sequence Detection System (Applied Biosystems, Foster City, CA). Primers specific for GFAP, NF-L, and Doppel were designed using the Primer Express program. Primer pairs used were as follows: GFAP forward (CTGGAGGTGGAGAGGGACAA), GFAP reverse (CAGCCTCAGGTTGGTTTCATCT), NF-L forward (CCGGCCGCCACCAT), NF-L reverse (CCACATAGCGCCGCTTGTA), Dpl forward (GCTGGTGGGCAAAGGTAGAC), Dpl reverse (TGAAACGCTACACGTTGTACTTTCA). Data were normalized to GAPDH expression using forward (GGTGGACCTCATGGCCTACA) and reverse (AGGGCCTCTCTCTTGCTCAGT) primers.

### Cerebellar granule neuron (CGN) cultures

CGNs were cultured from wild-type C57BL/6J × CBA/J mice or from *Prn-p*^*0/0 *^mice [3]. Cultures were prepared from 5-day-old mouse pups as described previously [35]. Neurons were suspended in K25+S medium (Basal Media Eagle with Earle's salts without glutamine, 10% dialyzed FCS, 2 mM glutamine, 25 mM KCl, and 0.02 mg/ml gentamicin) and plated in chamber slides coated with poly-D-lysine at a density of 560,000 cells/cm^2^. Two hours after plating, the medium was changed to K25 medium (no serum) supplemented with B27 (Invitrogen). Four days after plating, conditioned medium was removed and replaced with fresh K25+B27 medium. CGNs were co-transfected with 1 μg DNA (1:1 ratio of mouse Bax and pEGFP-N1 (Clontech, Mountain View, CA)) using Lipofectamine 2000. Mouse Bax cDNA was obtained from Open Biosystems (#MMM1013-64655; Huntsville, AL), PCR amplified, and cloned into the pcDNA3.1(+)/hygro plasmid (Invitrogen). After 2 hours, the neurons were returned to conditioned K25+B27 medium.

Transfected CGNs were fixed in 4% paraformaldehyde/5% sucrose in PBS for 10 minutes at room temperature. Nuclei were stained with DAPI for 10 minutes. Death of EGFP-positive neurons was determined morphologically based on chromatin condensation, degeneration of neurites, and cell body shrinkage. At least 100 neurons were individually scored for each experiment. Cells were imaged using a Nikon OptiPhot-2 microscope and MetaMorph imaging software.

CGNs were subjected to reduced extracellular potassium (5 mM) and serum deprivation as previously described [35]. Following this manipulation, neurons were washed twice with Locke's buffer and incubated for 10 minutes in 5 μM calcein AM (Invitrogen) in Locke's buffer at 37°C. CGNs were washed once with Locke's buffer and lysed in PBS/0.1% Triton-X100 followed by quantitation of the fluorescence signal on a microplate fluorimeter (Bio-Tek). Experimental values were expressed as a percentage of the calcein AM fluorescence values in K25+S control samples at each timepoint.

## Abbreviations

CGN: cerebellar granule neuron; Dpl: Doppel; EGFP: enhanced green fluorescent protein; GAPDH: glyceraldehyde-3-phosphate dehydrogenase; GFAP: glial fibrillary acid protein; LDH: lactate dehydrogenase; MTT: 3-(4,5-dimethyl-2-thiazolyl)-2,5-diphenyl-2H-tetrazolium bromide; NF-L: neurofilament light chain; PG14: nine-octapeptide insertional mutation in PrP; PrP: prion protein; PrP^C^: cellular isoform of PrP; PrP^Sc^: scrapie isoform of PrP; Tg: transgenic; TNF-α: tumor necrosis factor-α; TRAIL: tumor necrosis factor-related apoptosis inducing ligand.

## Competing interests

The authors declare that they have no competing interests.

## Authors' contributions

HC and DH conceived of the study, designed experiments, analyzed data, and wrote the manuscript. HC performed experiments. All authors read and approved the final manuscript.
